# Compass Fundus-Guided Perimetry in Geographic Atrophy

**DOI:** 10.1155/2022/1315588

**Published:** 2022-09-10

**Authors:** Roberta Farci, Arturo Carta, Paolo Fogagnolo, Luca Mario Rossetti, Maurizio Fossarello

**Affiliations:** ^1^Department of Health Science, University of Milan, Milan, Italy; ^2^Department of Surgical Science, Eye Clinic, University of Cagliari, Cagliari, Italy; ^3^Ophthalmology Unit, Department of Medicine and Surgery, University of Parma, Via Gramsci 14, Parma 43125, Italy; ^4^Eye Clinic and Head and Neck Department, ASST Santi Paolo e Carlo Hospital, University of Milan, Milan, Italy

## Abstract

**Purpose:**

To evaluate compass (CMP), a recently introduced device that combines scanning ophthalmoscopy, automated perimetry, and eye tracking, for fundus-guided perimetry (microperimetry) with the purpose of correlating perimetric retinal sensitivity (PRS) and retinal geographic atrophy (GA) features.

**Materials and Methods:**

A retrospective, cross-sectional study was performed in 56 eyes of 43 patients affected by GA. All patients underwent compass 10-2 perimetry, consisting of a full-threshold visual field on fundus photography and an infrared (IR) image of the central 30° of the retina. Data were exported to an Excel sheet. Binarization with black/white (B/W) variables was applied on the compass photo fundus and matched with visual field scores. Patients underwent autofluorescence (AF) and IR images (Heidelberg, Germany): CMP and Heidelberg IR images were homologated by using GIMP software (https://www.gimp.org), and then atrophic areas were manually measured with the ImageJ program. CMP perimetric grid was overlapped with AF and IR pictures by using GIMP, obtaining composite TIFF images, which were then analyzed with the ImageJ greyscale score (GSS) tool. A hyperautofluorescent halo was identified on the GA edges of some patients. Pearson's correlation between GA size on IR compass and IR Heidelberg and between GSS and PRS values has been calculated; the independent *t*-test was realized to calculate the correlation between GSS and B/W variables identified on the CMP photo fundus. The Spearman correlation between total deviation and pattern deviation was calculated.

**Results:**

The AUC-ROC score between CMP scores and B/W variables was 93,4%. The Spearman correlation between total deviation and pattern deviation was highly significant (*p* = 0,00). The correlation between AF GSS values and PRS was significant (*p* value = 0,00), the correlation between GSS of hyperautofluorescent points and PRS was significant (*p* value = 0,00), and the correlation between IR GSS and PRS was significant (*p* value = 0,00). The correlation between AF GSS and B/W variables was significant (*p* value = 0,002), the correlation between hyperautofluorescent points and B/W was not significant (*p* value = 0,40), and the correlation between IR GSS and B/W was significant (*p* = 0,00).

**Conclusions:**

Based on our preliminary results, compass seems to be a reliable, quick, and safe device for the anatomical and functional study of GA. The direct visualization of the visual field on the fundus photography as a background allows a precise assessment and clinical monitoring of this disease.

## 1. Introduction

Geographic atrophy (GA) is a progressive and irreversible advanced form of age-related macular degeneration (AMD) [1] whose prevalence is estimated to be approximately 8 million people worldwide [2–5]. Atrophic areas initially appear in the perifoveal retina, and patients do not perceive visual problems since their vision is still preserved. Sometimes, this process forms an atrophic ring around the fovea, which can remain unaffected for several years, a phenomenon known as foveal sparing [6, 7]. In this case, retinal fixation remains central and stable [8]. Previous studies demonstrated that GA lesions were found to enlarge at rates between 0.53 and 2.6 mm^2^/year [9–11].

There is currently no treatment available that can halt or reverse the progression of GA [12]. However, reliable diagnostic methods to monitor the progression of GA are of considerable importance to evaluate the efficacy of potential therapeutic agents, concerning both the structural and functional changes.

The anatomical-functional correlation on GA areas is rather unexplored [13–16]: standard methods for the diagnosis and the follow-up of GA are visual acuity, contrast sensitivity, fundus photography, AF and IR images, and optical coherence tomography (OCT). Microperimetry, or fundus-controlled perimetry, is a visual function test that provides a pointwise map of the retinal sensitivity. Microperimetry is currently the clinical investigation of choice to assess residual visual function in macular degenerative diseases, especially GA [17–20]. Nowadays, microperimetry is the most reliable diagnostic method to establish the function-structure relationship of GA; however, microperimetry devices currently available on the market are not equipped with a fundus photo as a background. The aim of this study was to evaluate a new instrument, called Compass (CMP) which is a fully automated device consisting of a scanning ophthalmoscope, collecting color fundus photos, IR, and red free of the posterior pole combined with an automated perimeter and an eye tracker.

The aim of this study was to assess the agreement of GA areas with fundus photography, AF, and IR images and to calculate the correspondence between retinal images and perimetric sensitivity.

Study objectives were as follows:

1. To evaluate the agreement of the following images to assess GA morphology: CMP true-color photography, CMP IR, Spectralis IR, and Spectralis AF;
2. To quantify the correspondence between PRS and the following images: CMP true-color photography, CMP IR, Heidelberg IR, and Heidelberg AF;
3. To evaluate the performance of PRS in AF areas;
4. A correlation between CMP, PRS, and this AF region was calculated.

## 2. Material and Methods

This study was conducted at the Eye Clinic of San Paolo Hospital, University of Milan, Italy, from April 2018 to June 2019. It has been conducted in compliance with the requirements of the Declaration of Helsinki .

**Figure 1:**
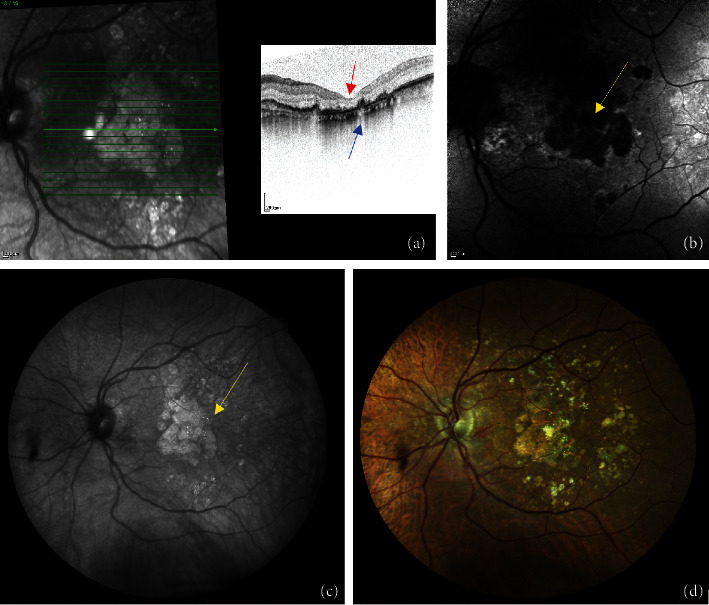
Multimodal imaging of GA as follows: (a) Heidelberg OCT of a GA shows the backscattering of the RPE, blue arrow, and the thinning of the IS/OS, red arrow; (b) AF of a GA lesion acquired with Heidelberg device, seen as hypofluorescent dark area, yellow arrow; (c) IR image of a GA taken with CMP shows GA as a white, hyper-reflective region, yellow arrow; and (d) photo fundus realized with CMP.

The study protocol was retrospective and cross-sectional. All patients underwent a complete ophthalmological examination, including measurement of the best-corrected visual acuity (BCVA) by means of Snellen eye chart (converting decimal acuity to logMAR), SD-OCT, AF, and IR imaging (Spectralis HRA + OCT, Heidelberg, Germany), and 10-2 CMP examination (Compass, CenterVue, Padova, Italy) consisting of a full threshold on the central 10° visual field superimposed to photo fundus and IR imaging. Both eyes, if eligible, were considered. —Figure 1.

Inclusion criteria were as follows: (1) to be affected by GA, characterized as a sharply shaped round or oval area of hypopigmentation, or absence of the retinal pigment epithelium (RPE), in which choroidal vessels are more visible than in surrounding areas, that must be at least 175 *μ*m in diameter [21], (2) age between 18 and 95 years, with BCVA logMAR ≥ + 0.1 (≥8/10 decimals) in the studied eye, (3) spherical refraction between −8D and +8D in the study eye; astigmatism between −2D and +2D in the study eye, and (4) normal optic nerve head in both eyes (no evidence of excavation, rim thinning, notching, disc hemorrhages, and RNFL thinning); (5) IOP less than 21 mmHg in both eyes.

Exclusion criteria were as follows: (1) BCVA <0.8 LogMAR, (2) presence of cataract C4 to C5 according to the Lens Opacities Classification System III, (3) history of choroidal neovascularization and treatment with anti-VEGF, (4) ocular surgery except for uncomplicated cataract surgery in both eyes performed less than 6 months before, (5) presence of pathologies that could affect visual field, and (6) use of drugs potentially interfering with the correct execution of perimetry.

Compass is a scanning ophthalmoscopy and automated perimetry, using a SLO system. Compass is able to take a photo fundus over a 30° radius field with a small pupil, hence no dilation is necessary for most subjects. IR images, acquired at the rate of 25 images per second, allow for continuous, automated, and tracking of eye movements. Determination of eye movements allows, in turn, active compensation of fixation losses, with perimetric stimuli being automatically repositioned before and during projection based on the current eye position. This mechanism reduces test-retest variability and ensures an accurate correlation between function (i.e., retinal threshold values) and structure (retinal appearance) [17, 18].

All tests were reviewed for the presence of artifacts, including inappropriate fixation, fatigue, inattention or learning effects, eyelid or rim artifacts, or any evidence of conditions other than GA affecting the results. Only reliable tests were selected. For CMP, reliability was FP ≤ 18%, BS ≤ 25%, and FN ≤ 30%; an average pupil diameter during the test >2,8 mm was respected.

### 2.1. Data Management

62 subjects fulfilled inclusion and exclusion criteria; 19 patients were excluded as follows: 10 for unstable fixation and 9 for unreliable CMP examinations.

### 2.2. Statistical Analysis

#### 2.2.1. Correlation between Retinal Images

Images of CMP with color photo fundus background were analyzed to evaluate the ROC curve.

CMP image was converted to black/white, with white dots for atrophic areas, and black for areas outside atrophic regions.

IR images (both at CMP and Spectralis) and AF images were homologated for each patient, by using the “Scale” tool with GIMP software (https://www.gimp.org). Thereby, the GA area was manually traced and the count of grey pixels (area) was performed using ImageJ (https://imagej.nih.gov) [22].

A Pearson correlation was calculated to compare the GA extent achieved with these two devices.

#### 2.2.2. Structure-Function Correlation

**Figure 2:**
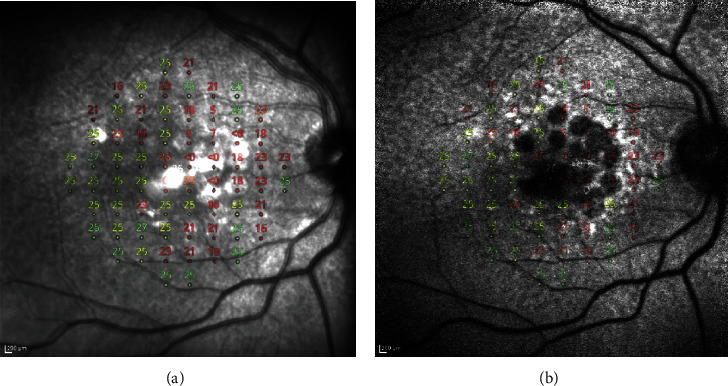
An example of a “merged image” obtained by using GIMP software, after overlapping a 10-2 CMP with Heidelberg IR (a) and AF (b) pictures.

In order to evaluate the agreement between structure (with any image) and PRS at CMP, the perimetric grid overimposed the images elaborated as described in the previous paragraph (Figure 2). TIFF images were obtained and analyzed with the ImageJ software by placing the cursor on each grid perimetric point and recording the correspondent GSS of the fundus image. The pointwise correlation between GSS and perimetric sensitivities was then calculated. Moreover, the data were exported to Excel to create the plots, and a binarization process was applied. B/W code was assigned relying on the observation of the points in correspondence with the AF and IR background. Total deviation points and pattern deviation percentages were also reported.

The analysis aimed to assess

(1) Whether the Compass PRS grid was predictive of a white value of the fundus background;
(2) Whether an agreement between GA areas, as measured both with IR CMP and IR Heidelberg, was present;
(3) Whether there was a correlation between the GSS and the CMP Total Deviation points and Pattern Deviation percentages (correlation index) after overlapping CMP with Heidelberg AF and IR images, respectively.
  1. A logistic mixed effect regression was performed [23]. The correlation analysis between the Total Deviation and Pattern Deviation was calculated using a Spearman nonparametric correlation analysis. The R Statistical software (R Core Team 2018) has been used to perform statistical analysis.
  2. GA areas were measured twice by the same operator, in order to assess the test-retest variability. A Pearson's correlation between Heidelberg and CMP measurements has been calculated; then, a Pearson's correlation between the intraobserver measurements with Compass and Heidelberg, respectively, has been performed.
  3. Concerning the correlation between Compass PRS and GSS on IR and AF images, a Pearson correlation between GSS and Total Deviation points and Pattern Deviation percentages test was performed. An independent sample test has been realized in order to evaluate the correlation between the GSS and the B/W regions both on AF and on IR background and between GSS and the hyperautofluorescent points on AF images.

K Cohen's agreement among CMP fundus photo, IR Heidelberg, AF Heidelberg, and IR Compass and among CMP IR and IR and AF Heidelberg, respectively, has been calculated.

## 3. Results

56 eyes of 43 participants were enrolled in this study. 30 were females and 13 males; age was 79±9 years and BCVA 0,5 ± 0,32 (LogMAR); MD was −8,54 ± 6,46 dB and PSD 7,72 ± 3,38 dB. Demographic characteristics of the patients are reported in Table 1.

### 3.1. Correlation between Retinal Images

#### 3.1.1. Correlation between GA Size using CMP and Heidelberg IR Images

The average of GA area as seen on CMP IR was 30,7 *μ*m^2^ ± 26,7 (measurement 1) and 31,4 *μ*m^2^ ± 26,9 (measurement 2); the average of the GA size as examined on IR Heidelberg was 41,0 *μ*m^2^ ± 30,7 (measurement 1) and 40,9 *μ*m^2^ ± 30,2 (measurement 2).

The correlation between CMP and Heidelberg measurements 1 was significant (*p*=0,01); the correlation between CMP and Heidelberg measurements 2 was significant (*p*=0,01); the correlation between CMP intraobserver tests was significant (*p*=0,00); the correlation between Heidelberg intraobserver exams was significant (*p*=0,00) Table 2.

### 3.2. Structure-Function Correlation

#### 3.2.1. Correlation between CMP PRS and Heidelberg AF Images

1173 measurements were made, of which 76,1% were black and 23,4% were white; 6 did not show any black or white value. The average of PRS was 20,9 ± 9,1; the average of pattern deviation was 3,5% ± 2,1; and the average of GSS was 121,5 ± 54,5.

A significant correlation between GSS and PRS and Pattern Deviation, respectively, was found (*p* value < 0,00). The correlation between GSS and the B/W variables was significant (*p* value = 0,00) Table 3.

#### 3.2.2. Correlation between GSS and Heidelberg Hyperautofluorescent Points

A significant correlation between GSS and CMP PRS and Pattern Deviation of the hyperautofluorescent edges was found (*p* value = 0,00). The correlation between hyperautofluorescent points GSS and the B/W variables was not significant (*p* value = 0,4) Table 3.

#### 3.2.3. Correlation between GSS and Heidelberg IR Images

1175 measurements were made, of which 18,6% were black and 80,9% were white, 6 of which did not show any black or white value.

The average of PRS was 21,6 ± 9,3, the average of Pattern Deviation value was 3,6% ± 2,0%, and the average of GSS was 155,7 ± 49,3. A significant correlation between GSS and Total Deviation and Pattern Deviation was found (*p* value < 0,001). The correlation between GSS and the B/W variables was negative and significant (*p* value = 0,002) Table 3.

#### 3.2.4. Correlation between CMP PRS and Fundus Photo

**Figure 3:**
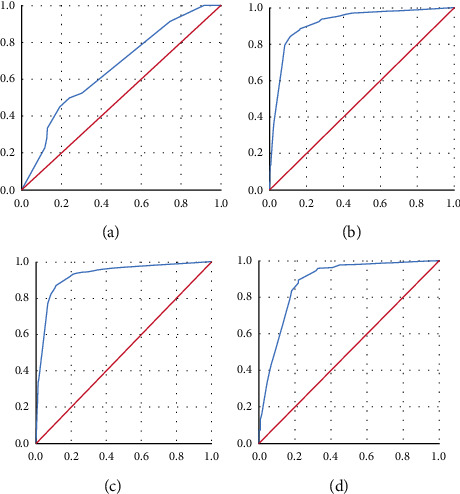
The AUC-ROC score and corresponding confidence intervals are shown in the plot as follows: AOU score of 0,917 between CMP fundus photo and CMP perimetric scores (a) indicates a very high sensitivity and specificity of CMP to individuate retinal atrophic regions. The AOU-roc curve between CMP perimetric values and CMP IR was 0,926, (b) the AOU-roc curve between CMP perimetric scores and AF Heidelberg was 0,663, and (c) the AOU-roc curve between CMP and IR Heidelberg was 0,882 (d).

The AOU-roc score between CMP and fundus photo was strongly significant at 0,917. The AOU-roc curve between CMP perimetric values and CMP IR was 0,926, the AOU-roc curve between CMP perimetric scores and AF Heidelberg was 0,663, and the AOU-roc curve between CMP and IR Heidelberg was 0,882 (Figure 3). The correlation between Total Deviation and Pattern Deviation was positive and significant (*p* = 0,00).

K Cohen between CMP fundus photo and CMP IR was 0,739, K Cohen between CMP fundus photo and AF Heidelberg was −0,132, K Cohen between CMP photo fundus and IR Heidelberg was 0,714, K Cohen between CMP IR and AF Heidelberg was −0,133, and K Cohen between CMP IR and IR Heidelberg was 0,841 (Table 4).

The average of false positives was 2,1 ± 11,0, the average of false negatives was 8,0 ± 8,9, the average of fixation loss was 0,04 ± 0,7, the average of pupil size was 4,3 ± 1,7 mm, the average of duration was 8,9 ± 2,5 minutes, and the average of fovea sensitivity was 25,4 ± 8,3. Forty-nine eyes showed foveal sparing: 7 eyes showed the absence of foveal sparing.

We tested a 10° field which corresponded to 3000 *μ*m in diameter and 14,15 mm^2^ in area, and the approximate area covered by each tested point was 14,15/34 = 0,21 mm^2^.

## 4. Discussion

Since the kinetics of GA progression are highly variable among individual patients, there is no currently systematic method for rating an individual's GA severity that incorporates various lesion characteristics, particularly those affecting visual function. As discussed earlier, tests such as BCVA are not reliable for patients with GA, especially for those with foveal sparing; AF or OCT provide misleading information since they do not provide functional information. All the currently available tests do not allow for a correlation between anatomy and functional outcomes. Compass 10-2 with color photography of the fundus or IR as a background provides a reliable visualization pointwise of the macular retinal sensitivity, consenting to match, point by point, sensitivity values, expressed in Decibel, to the retinal image [24, 25].

In the present work on patients affected by GA, we matched GSS points calculated from 10-2 Compass perimetry with AF and IR images obtained with Heidelberg retinography.

In particular, we focused our attention on the perilesional areas bordering GA, the so-called junctional lesion, which represents a critical area to monitor the enlargement of retinal atrophy. This area appears to be hyperautofluorescent due to the excessive accumulation of lipofuscin in RPE cells and is associated with lower sensitivity than in normal retinas [26].

In our study, we individuated the PRS points adjacent to the GA area with Compass, and we matched them to visualize them on the AF and IR retinal images, in order to verify the correspondence. We found a significant correspondence between the GSS of AF and IR images and Total Deviation and Pattern Deviation, respectively, of the Compass perimeter.

Patients without foveal sparing utilize photoreceptors neighbouring the GA lesion to fixate the target. In this study, we found that Compass Eye Tracker provides a fixation method that agrees with previous measurement methods [17, 27–29] with perimetric stimuli being automatically repositioned before and during projection based on the current eye position. This mechanism reduces test-retest variability and ensures an accurate correlation between function (i.e., retinal threshold values) and structure (retinal appearance).

Sayegh et al. found that stimulation points with a sensitivity of more than 1 dB were found mainly in the borders of the area of atrophy [30]. In a study conducted by Hartmann et al., sensitivity tests in patients with GA have been realized: a mean sensitivity of 5,29 ± 2,49 dB at the margin of the lesion was discovered in comparison with 14 ± 2,4 dB in the area of the uninvolved retina [31].

Meleth et al. identified perilesional and extralesional points around the GA region. They found that perilesional points, corresponding to the junctional zone, were consistently less sensitive than extralesional points in multiple microperimetry assessments and declined in sensitivity more rapidly with follow-up time than for extralesional points [32]. The retinal sensitivity in the areas outside the GA lesion was significantly lower than those found in equivalent areas in healthy subjects of the same age, and the MS, after having matched all the corresponding points, was significantly reduced as a function of time. This result indicated that functional waning in GA corresponds not only to an anatomic increase in the size of an absolute scotoma but also a general decrease in macular sensitivity in the tested area. They speculated that it may due to a more general loss of macular sensitivity in areas around the GA lesion since it occurs as a separate process from the local expansion of the GA lesion itself.

Chen et al. repeated microperimetry at baseline, 6 months, and 12 months to patients affected by GA who reported no significant changes in visual acuity, fixation stability, and macular sensitivity over 1 year. A significant reduction in MS within the macular region and test loci adjacent to dense scotoma was found [33]. So that, retinal points around GA represent an index of progression. Similarly, we found a significant correlation between hyperautofluorescent border points and Compass PRS, that is to say, that Compass could be considered as accurate as microperimetry for studying retinal junctional lesions.

This study presents some limitations as follows: a relatively small number of participants, the lack of a follow-up, we have also not quantified the test-retest variability in our patient population, and Compass does not allow to see AF as a background to identify junction hyperfluorescent signal, in order to match visual field points.

In conclusion, this pilot study suggests that Compass may represent a valuable tool to perform a structural-functional analysis of GA features and can be used in the management of these patients to evaluate the follow-up.

## Figures and Tables

**Table 1:** Demographic characteristics of patients.

	Total
Age	79,31±9,3
Caucasian	41
Hispanic	1
East European	1
Right/left	29/27
Female/male	30/13
Best-corrected visual acuity	0,48 ± 0,33

**Table 2:** Pearson's correlation between intraobserver GA measurements (measurements 1 and 2) and between CMP and Heidelberg IR imaging.

GA area	Heidelberg measurement 1	Compass measurement 2
Heidelberg measurement 2	*R* = 0,99 *p*=0,00	*R* = 0,69 *p*=0,00
Compass measurement 1	*R* = 0,56 *p*=0,01	*R* = 0,99 *p*=0,00

**Table 3:** Correlations between AF, hyperautofluorescent points, and IR grey scales with CMP PRS, CMP Pattern Deviation, and B/W, respectively.

	*n*	PRS	Pattern deviation	B/W
GSS on AF	1167	*R* = 0,28 *p*=0,000	*R* = 0,15 *p*=0,000	*R* = 0,02 *p*=0,00
GSS of hyperautofluorescent points	184	*R* = 0,14 *p*=0,06	*R* = 0,05 *p*=0,00	*R* = 0,42 *p*=0,45
GSS on IR	1170	*R* = −0,15 *p*=0,000	*R* = −0,13 *p*=0,000	*R* = 0,00 *p*=0,00

**Table 4:** K Cohen correlation between all the variables.

	Compass IR	AF Heidelberg	IR Heidelberg
Compass fundus photo	0,739	−0,132	0,714
Compass IR		−0,133	0,841

## Data Availability

The authors confirm that all data underlying the findings in the present study are freely available in the manuscript.
